# Sexual Health in Spanish People with Intellectual Disability: the Impact of the Lockdown due to COVID-19

**DOI:** 10.1007/s13178-021-00621-7

**Published:** 2021-07-24

**Authors:** M. Dolores Gil-Llario, Irene Díaz-Rodríguez, Vicente Morell-Mengual, Beatriz Gil-Juliá, Rafael Ballester-Arnal

**Affiliations:** 1 Department of Developmental and Educational Psychology, University of Valencia, Valencia, Spain; 2 Department of Personality, Evaluation and Psychological Treatment, University of Valencia, Valencia, Spain; 3 Department of Basic and Clinical Psychology and Psychobiology, Jaume I University, Castellon, Spain

**Keywords:** COVID-19, Spain, Lockdown, Impact, Sexuality, Intellectual disability

## Abstract

**Introduction:**

The lockdown due to COVID-19 affected the sexual health of the people with intellectual disabilities by differentially modifying the frequency and characteristics of people’s sexual activity depending on whether or not they lived with a partner during this period. The aim of this study was to analyze the extent to which the sexual behavior of people with intellectual disabilities (with and without a partner) was affected during the lockdown.

**Methods:**

The sample consisted of 73 people with intellectual disabilities between 21 and 63 years old (M = 39.63; SD = 10.11). The variables analyzed were the physical, social, and technological environment during the lockdown, sexual appetite, sexual behavior, online sexual activity, and sexual abuse. The data were collected between the months of May and June of 2020.

**Results:**

The lockdown increased the sexual appetite of a third of the sample (38%), especially the youngest participants. Sexual activity focused on autoeroticism and online behavior, particularly sending nude images of oneself (88%) and viewing pornography (83.6%). Rates of sexual abuse during this period were relatively high (6.8%).

**Conclusions:**

The sexual activity of people with ID was important during the lockdown, and they had to adapt to the circumstances of isolation in a similar way to the general population. Technological improvements in terms of devices and connection quality at home allowed their sexual behavior to be reoriented, opening the door to new risks for the sexual health of people with ID.

**Policy Implications:**

Cybersex and the increase in sexual abuse due to confinement are aspects that should be included in programs to improve the sexual health of this group.

## Introduction

The sexuality of people with intellectual disability has traditionally been a controversial issue (Franco et al., 2012). The medical model that prevailed for a long time was based on a series of myths that justified the annulment of their experience of sexuality (Jiménez & Huete, 2013). In some cases, their sexuality was equated with that of children, with an absence of sexual desire (Borawska-Charko et al., 2017). In other cases, a lack of control over their impulses was assumed, justifying the prohibition of any expression of their sexuality in order to protect themselves and others (Rodríguez et al., 2006). The overprotective and paternalistic perspective has been replaced by the current models focusing more on providing support on those aspects where is needed rather than imposing prohibitions (Arnau, 2019). There has been an increase in the number of studies designed to determine their demands and needs in order to create programs that provide the necessary support to allow them to fully and responsibly experience their sexuality. Thus, in recent years, professionals who care for people with intellectual disability in occupational or residential centers have asked to receive training in affective-sexual education. These improvements, which have had an impact on the quality of life of people with ID, have not occurred in a generalized way in this group. Instead, they depend on the degree to which their guardians, whether parents in the family unit or professionals in residential centers, have adopted this normalizing perspective (Hoyos & García, 2017). This is a slow process because, especially in the case of parents, it involves people from previous generations (parents of adults with ID are usually over 60 years old) who have grown up with much more restrictive values as far as sexuality is concerned, even in the general population (Manor-Binyamini & Schreiber-Divon, 2019).

In this context of slow and uncertain progress in terms of the sexuality of people with ID, and recognizing that they only have the sexuality they are allowed to have, it is worth asking to what extent people with ID have been affected by the restrictions on mobility and contact with others adopted by many governments, among others, in response to the terrible COVID-19 pandemic.

### Sexuality in People with Intellectual Disability

However, before analyzing the implications of the lockdown, let us briefly review what we know about the sexuality of people with ID. Recent reliable and rigorous studies reveal that people with ID show interest in sexuality from very early ages, with sexual desires and needs similar to those shown by the general population (Borawska-Charko, et al., 2017; Gil-Llario, 2019). A study conducted by Gil-Llario et al. (2018) found nearly all adults with ID are interested in sexuality and 96.4% have had a partner. When they do not have a partner, most people with ID express their desire to have one (Gil-Llario et al., 2018) or to have an intimate relationship (Lafferty et al., 2013; Turner & Crane, 2016).

As far as their sexual activity is concerned, between 73 and 85% of people with ID have engaged in sexual intercourse (Baines et al., 2018; Díaz-Rodríguez et al., 2014; Morell-Mengual et al., 2016), with the percentage being higher in women from 40 to 55 years old (Gil-Llario et al., 2018). Higher percentages have been found for masturbation (76–92%), with this sexual practice being considered the most frequent one in this population group, especially in men (Díaz-Rodríguez et al., 2014; Kijak, 2013).

As we have seen, this is a group with a high level of sexual activity, but it is worth asking to what extent this activity is desired and voluntary or, unfortunately, occurs in a context of coercion. The available data on sexual abuse in people with ID, although inconsistent due to different factors such as the criteria established to determine it, cultural differences, etc., (Gil-Llario et al., 2018; Tomsa et al., 2021), reveal high abuse figures in this group. Moreover, there is some discrepancy between professionals’ awareness of the experience of sexual abuse and the reality reported by the people who have experienced it (Giménez-García et al., 2017). When the data are self-reported, we find percentages of around 9% for women and 3% for men (Gil-Llario et al., 2019; Mitra et al., 2016), but these percentages increase considerably when reported by professionals (24–28% in women and 16–30% in men) (Gil-Llario et al., 2019; Haydon et al., 2011; Swango-Wilson, 2009). This prevalence could be even higher, given the difficulty people with ID have in identifying sexual abuse. The lack of self-reporting of sexual abuse could be explained by several factors, such as cognitive limitations, communication problems, difficulties in distinguishing between public and private, embarrassment, and negative reactions from the environment when the event is reported. The latter is not a trivial problem because, unfortunately, most cases of abuse are carried out by people close to them who are responsible for their education and care (Diaz-Rodriguez et al., 2014; Martinello, 2014; Medina-Rico et al., 2018). In this regard, recent research shows deficiencies in affective and sexual education in this group, in terms of both conceptual aspects and skills, which makes it difficult to identify indicators of risk of abuse, thus making people with ID much more vulnerable (Byrne, 2018; Gil-Llario, 2019; Sinclair et al., 2015). According to the WHO (2021), children with ID present a risk of sexual abuse 4.6 times greater than their peers without ID.

### Sexual Behavior During Lockdown

But what are the impact of COVID-19 on Sexuality and Disability: Are We Closer or More Isolated? Ask Sigmund Hough (2020) in a recently published editorial. Environmental and psychological adverse conditions affect sexual functioning and sexual behavior. Isolation may have made their already complicated sexual interactions more difficult to a greater extent. However, there is a lack of studies designed to analyze the impact of the pandemic on the sexual health of people with intellectual disability (Lokot & Avakyan, 2020).

The context of confinement has highly likely further limited the sexual rights and freedoms among people with ID, a sexually active but highly vulnerable group with restricted access to sexuality (Lokot & Avakyan, 2020). They are routinely forced to develop their sexuality and relationships in a framework of dependency, with a narrow social and private life and significant restrictions (Frawley & Wilson, 2016) that become much more evident during a lockdown like the one caused by COVID-19. This scenario of home lockdown or confinement in residences has deprived people with ID of contact with people other than cohabitants or caregivers, following strict recommendations for social distancing. In turn, the lockdown has contributed to further reducing the limited privacy people with ID tend to have (Rogers, 2009), which can often be almost none. All of this is combined with the existing conservative attitudes about the sexuality of people with ID, especially those held by immediate family members (Bazzo et al., 2007; Morell-Mengual et al., 2017; Tamas et al., 2019), and prohibitions imposed by overprotective parents, or in the case of residential centers, restrictive rules that limit the control people with ID have over their lives (Murphy & Bantry-White, 2020). This restrictive context may have influenced their sexual interest or desire, as well as the actual possibilities of carrying out sexual practices, not only with a partner, but also alone.

Likewise, it is important to keep in mind that, in the best-case scenario, and when a certain degree of privacy has been guaranteed, the situation of confinement may also have led to changes in the repertoire of sexual behaviors, with other sexual activities, such as those carried out online, becoming more important. Although this is still a relatively unexplored area, a growing number of studies show the use of new technologies by people with ID (Chiner et al., 2017; Ramsten et al., 2020). Along these lines, one of the few studies that has explored access to social networks by this group was carried out in Australia. It reveals that the main uses of social networks include making new friends, maintaining existing friendships, and exploring and expressing sexuality (Darragh et al., 2017).

Another consequence of confinement is the increase in the levels of sexual abuse, an aspect observed in the general population, based on the increase in the number of calls for help to services for abuse victims. The lockdown that people with ID have had to experience for months may have made it more difficult to identify situations of risk of sexual abuse, and it may even have aggravated these situations because many cases of abuse occur in the family context (Gil-Llario, 2019; Hughes et al., 2012).

### Current Study

For all these reasons, the aim of this study was to analyze the experience of sexuality of people with ID during the period of confinement due to COVID-19. For this purpose, we analyzed the physical, social, and technological environment where these people were confined, as well as the sexual desire, sexual behavior, practice of online sexual behaviors, and presence of sexual abuse. These aspects were analyzed taking into account variables such as gender, age, whether they had a partner, and the availability of electronic devices.

## Method

### Participants

The sample consisted of 73 adults with mild intellectual disability (*n* = 43 men and *n* = 30 women) between 21 and 63 years old (M = 39.63; SD = 10.11); 16.4% were between 21 and 29, 37% between 30 and 39, 30.1% between 40 and 49, 11% between 50 and 59, and 5.5% were more than 60 years old. All the participants were receiving some type of community health resource, either in an occupational center or in a care center. Regarding their sexual orientation, 83.5% identified themselves as heterosexual (79% men and 90% women), 11% as homosexual (14% men and 6.7% women), and 5.5% as asexual (7% men and 3.3% women). A total of 48.8% of the men and 53.3% of the women reported having a stable partner. Table 1 presents the sociodemographic characteristics by gender in greater detail.

**Table 1:** Participants’ characteristics

	Total (*n* = 73) % or M (SD)	Men (*n* = 43; 58.9%) % or M (SD)	Women (*n* = 30; 41.1%) % or M (SD)
Age			
Mean age	39.63 (10.11)	39.84 (9.65)	39.33 (10.90)
Between 21 and 29 years old	16.4%	14%	20%
Between 30 and 39 years old	37%	44.2%	26.7%
Between 40 and 49 years old	30.1%	25.6%	36.7%
Between 50 and 59 years old	11%	11.6%	10%
Between 60 and 63 years old	5.5%	4.6%	6.6%
Sexual orientation			
Heterosexual	83.5%	79.1%	90%
Homosexual	11%	14%	6.7%
Asexual	5.5%	7%	3.3%
Steady partner			
Yes	50.7%	48.8%	53.3%
No	49.3%	51.2%	46.7%

### Measures

An ad hoc questionnaire composed of 18 items with different response formats was used to assess different facets of sexuality developed during the confinement period due to COVID-19. The instrument is an adaptation of the one used in a parallel investigation carried out with the general population (Ballester-Arnal et al., 2020). The following areas were evaluated:

- **Physical, Social, and Technological Environment Where the Lockdown Took Place.** This section consists of six items that evaluate whether, during the confinement period, the person was in a residential center or with the family and, in the latter case, the type and number of people with whom he/she lived and whether they are the people with whom he/she usually lives. Information is also collected on the availability of a room of their own where they could engage in sexual activity without being disturbed or interrupted, as well as the possibility of having access to electronic devices at any time of day or night.
- **Sexual Appetite.** The intensity of their sexual appetite during lockdown was evaluated with a single item that collects the subjective rating: “How intense was your sexual appetite or desire during the lockdown?.” This question was answered on a Likert-type scale with three response options ranging from “less intense than before” to “more intense than before.”
- **Sexual Behavior.** This section consists of four items that collect information about different sexual practices carried out during the lockdown: masturbation and sexual relations. The items have a dichotomous response format “Yes” or “No.” If they refer to masturbation, there is an additional item that explores the type of masturbation: traditional or manual, with sex toys or watching pornography. If they refer to having sexual relations, the items explore whether they occurred in a physical context or online.
- **Other Sexual Contexts Online.** The practice of online sexual behaviors during the lockdown was evaluated through five items with a dichotomous response format: “I have looked for information about sex education,” “I have seen pornographic images or films,” “I have entered dating websites,” “I sent nude pictures of myself,” and “others.”
- **Sexual Abuse.** This section consists of two items that ask whether they have been forced to have sexual relations or whether they have forced someone else to have sexual relations during the lockdown period. The item responses have a dichotomous format “Yes” or “No.”

### Procedure

On March 14, 2020, the Spanish Government declared a state of alarm to manage the health crisis due to COVID-19 (Decree 463/2020), limiting the freedom of movement of people until May 2nd, at which time outings for walking or physical activity were allowed in different time slots according to the age range in towns with more than 5000 inhabitants. Finally, the state of alarm ended on June 21st.

After the lockdown ended, a descriptive study was designed based on the study conducted by Ballester-Arnal et al. (2020). Responses were collected using convenience sampling among users of an occupational center for people with intellectual disabilities located in the province of Cadiz (Spain). To determine the participants’ eligibility, the following inclusion criteria were established: (a) being over 18 years of age; (b) meeting the criteria of the Diagnostic and Statistical Manual of Mental Disorders for Mild Intellectual Disability (American Psychiatric Association, 2013); and (c) having sufficient communication skills to answer the different questions. The assessment was conducted by the psychologist of the occupational center in interview format and online via videoconference, between the months of May and June of 2020. The average time per assessment was 40 min, 10 of which were devoted to establishing rapport with the participant. The research had the approval of the Deontological Commission of the University of Valencia, and the ethical principles of the Declaration of Helsinki were followed at all times.

### Statistical Analyses

Data analysis was performed with the statistical software IMB SPSS Statistics 24. The environment where the confinement took place, sexual appetite, sexual behavior, and sexual abuse were compared based on gender (male or female), age (between 20 and 35 years or between 36 and 60 years), marital status (with or without a partner), and availability of electronic devices (yes or no). The different categorical variables were compared using a chi-square test, and Cramer’s *V* coefficient was used to calculate the effect size.

## Results

### Physical, Social, and Technological Environment Where the Lockdown Took Place

As Table 2 shows, most of the individuals evaluated spent the months of confinement with their family (87.7%), compared to 12.3% who were in a residential center, with no statistically significant differences based on gender or age range. In the family environment, the people with whom they mainly lived were the father or mother (71.2%) and the brother or sister (54.8%). Although men and women were generally accompanied by the same people, a higher percentage of men were accompanied by their parents (79.1%), compared to women (60%). It is also noteworthy that care provided by a younger relative (niece or nephew) was only the case for men between 36 and 60 years of age. In addition, almost two-thirds of the participants reported having their own room (64.4%). Finally, 54.8% of the sample, with no gender or age differences, had free access to electronic devices at any time of day or night. Thus, taking into account that the percentages are cumulative, and that the majority had access to more than one device, 97.3% had a cell phone, 61.1% had a computer, and 45.2% had a tablet.

**Table 2:** Physical, social, and technological variables associated with the confinement context

		Total (*n* = 73)	M (*n* = 43)	W (*n* = 30)	*X*^2^	*V*	20–35 (*n* = 27)	36–60 (*n* = 46)	*X*^2^	*V*
Type of confinement	Residence	12.3%	14%	10%	0.256	0.059	11.1%	13%	0.059	0.028
	Family	87.7%	86%	90%			88.9%	87%		
If confined with family …	Parent	71.2%	79.1%	60%	3.136	0.207	70.4%	71.7%	0.016	0.015
	Brother/sister	54.8%	53.5%	56.7%	0.072	0.031	63%	50%	1.154	0.126
	Brother/sister-in-law	9.6%	9.3%	10%	0.010	0.012	11.1%	8.7%	0.114	0.040
	Nephew/niece	6.8%	11.6%	0%	3.745	0.226	0%	10.9%	3.151	0.208
	Caregiver	5.5%	7%	3.3%	0.453	0.079	3.7%	6.5%	0.261	0.060
	Grandparent	5.5%	4.7%	6.7%	0.139	0.044	11.1%	2.2%	2.624	0.190
	Aunt/uncle	4.1%	2.3%	6.7%	0.845	0.108	3.7%	4.3%	0.018	0.016
Own room	Yes	64.4%	60.5%	70%	0.701	0.098	55.6%	69.6%	1.456	0.141
Electronic devices	Yes	54.8%	53.2%	56.7%	0.072	0.031	59.3%	52.2%	0.345	0.069
Type of device…	Cell phone	97.3%	97.7%	96.7%	0.067	0.030	100%	92.6%	3.503	0.219
	Tablet	45.2%	48.8%	40%	0.557	0.087	47.8%	40.7%	0.345	0.069
	Computer	61.6%	62.8%	60%	0.058	0.028	66.7%	58.7%	0.457	0.079

*M* man; *W* woman

### Sexuality and Sexual Behavior by Gender

Regarding the intensity of their sexual appetite (see Table 3), men experienced greater sexual appetite during lockdown than women, although the differences between the two groups were not significant (*p* = 0.436, *V* = 0.153). All participants, regardless of gender, reported having masturbated during confinement. The most common type of masturbation performed was traditional or manual masturbation (100% in both men and women), followed by masturbation while viewing pornography (23.3% in men and 20% in women). Overall, women reported significantly higher rates of masturbation with sex toys (*p* = 0.001), with a moderate effect size (*V* = 0.396). The online sexual behaviors performed most varied by gender; however, the only behavior that reached statistical significance was sending nude images of oneself, where men reported higher rates than women (100% vs. 75%; *p* = 0.050; *V* = 0.384). The percentage of men and women who claimed to have felt forced sexually was roughly equivalent. In contrast, when they recognized having forced themselves on another person, the percentage of men was significantly higher than that of women, and these differences were statistically significant (*p* = 0.033) with a small effect size (*V* = 0.265).

**Table 3:** Sexuality and sexual behavior by gender

		Total (*n* = 73)	Man (*n* = 43)	Woman (*n* = 30)	*X*^2^	*V*
Intensity of sexual appetite	Less	38%	31.7%	46.7%	1.658	0.153
	The same	23.9%	26.8%	20%		
	Greater	38%	41.5%	33.3%		
Masturbation (yes)	Traditional	100%	100%	100%	–	–
	Sex toys	13.7%	2.3%	30%	11.448^***^	0.396
	Pornography	21.9%	23.3%	20%	0.109	0.039
Sexual relations with other people (yes)	Physical	5.5%	7%	3.3%	0.453	0.079
	Online	4.1%	4.7%	3.3%	0.078	0.033
Online sexual behaviors (yes)	Search for sexual information	21.9%	16.3%	30%	1.944	0.163
	Watch pornographic films	83.6%	86%	80%	0.470	0.080
	Enter dating websites	12.3%	11.6%	13.3%	0.048	0.026
	Send nude images of oneself	88%	100%	75%	3.693^*^	0.384
Sexual abuse (yes)	Feeling forced	6.8%	7%	6.7%	0.033	0.006
	Forcing someone else	19.2%	27.9%	6.7%	5.143^*^	0.265

^*^p < .05; ^***^p < .001

### Sexuality and Sexual Behavior by Age

The intensity of their sexual appetite during confinement varied according to the age range (see Table 4). Thus, individuals between 20 and 35 years of age reported a greater sexual appetite (63%) than individuals between 35 and 60 years of age (22.7%). These differences were statistically significant (*p* = 0.003), with a moderate effect size (*V* = 0.408). In general, and in both age ranges, the most common sexual practices were traditional or manual masturbation, followed by masturbation while viewing pornography, masturbation with sex toys, physical sex, and online sex. However, significant differences were only found in masturbation behavior while viewing pornography, in favor of young people between 20 and 35 years of age (*p* = 0.002; *V* = 0.208). Finally, it is worth noting that no young people between 20 and 35 years of age reported feeling sexually forced by another person, compared to 10.9% of those between 36 and 60 years of age, although these differences did not reach statistical significance (*p* = 0.150, *V* = 0.208).

**Table 4:** Sexuality and sexual behavior by age group

		Total (*n* = 73)	Young people (*n* = 27)	Older people (*n* = 46)	*X*^2^	*V*
Intensity of sexual appetite	Less	38%	25.9%	45.5%	11.798^**^	0.408
	The same	23.9%	11.1%	31.8%		
	Greater	38%	63%	22.7%		
Masturbation (yes)	Traditional	100%	100%	100%	–	–
	Sex toys	13.7%	18.5%	10.9%	0.842	0.107
	Pornography	21.9%	37%	13%	5.723^*^	0.208
Sexual relations with other people (yes)	Physical	5.5%	7.4%	4.3%	0.308	0.065
	Online	4.1%	7.4%	2.2%	1.183	0.127
Online sexual behavior (yes)	Search for sexual information	21.9%	22.2%	21.7%	0.002	0.006
	Watch pornographic films	83.6%	74.1%	89.1%	2.808	0.196
	Enter dating websites	12.3%	7.4%	15.2%	0.960	0.277
	Send nude images of oneself	88%	92.9%	81.8%	0.711	0.169
Sexual abuse (yes)	Feeling forced	6.8%	0%	10.9%	3.151	0.208
	Forcing someone else	19.2%	18.5%	19.6%	0.012	0.013

^*^p < .05; ^**^p < .01

### Sexuality and Sexual Behavior According to Marital Status

The intensity of sexual appetite was mediated by the marital status (see Table 5). In general, people with a stable partner increased their appetite during lockdown, whereas single people experienced a decline, but these differences were not statistically significant (*p* = 0.077; *V* = 0.277). Regardless of their marital status, everyone who was evaluated reported having masturbated during the confinement period. However, people who had a partner had higher percentages of masturbation with sex toys (*p* = 0.172, *V* = 0.191) and masturbation while viewing pornographic content (*p* = 0.044; *V* = 0.265). In addition, only those in a dating relationship engaged in sex physically (12.5%) or online (9.4%) during lockdown, although only the former type of sexual relation reached statistical significance (*p* = 0.044; *V* = 0.265). Online sexual behavior also varied depending on the marital status. People with a partner reported higher percentages of sending nude images of oneself (100% vs. 87.5%; *p* = 546) and viewing pornographic images or movies (87.5% vs. 77.8%; *p* = 0.353); and single people reported higher percentages of seeking sexual information (27.8% vs. 18.8%; *p* = 0.408) and looking for a partner on dating websites (16.7% vs. 6.3%; *p* = 0.266). Regarding sexual abuse, the percentage of people with a partner who admitted having forced themselves on another person was higher (25%) than for single people (16.7%), although these differences were not significant (*p* = 0.549, *V* = 0.103).

**Table 5:** Sexuality and sexual behavior according to marital status

		Total (*n* = 71)	Single (*n* = 36)	Partner (*n* = 35)	*X*^2^	*V*
Intensity of sexual appetite	Less	35.8%	44.4%	25.8%	5.137	0.277
	The same	23.9%	27.8%	19.4%		
	Greater	40.3%	27.8%	54.8%		
Masturbation (yes)	Traditional	100%	100%	100%	–	–
	Sex toys	14.7%	8.3%	21.9%	2.477	0.191
	Pornography	22.1%	11.1%	34.4%	5.333^*^	0.280
Sexual relations with other people (yes)	Physical	5.9%	0%	12.5%	4.781^*^	0.265
	Online	4.4%	0%	9.4%	3.531	0.228
Online sexual behavior (yes)	Search for sexual information	23.5%	27.8%	18.8%	0.767	0.106
	Watch pornographic films	82.4%	77.8%	87.5%	1.102	0.127
	Enter dating websites	11.8%	16.7%	6.3%	1.771	0.161
	Send nude images of oneself	87.5%	83.3%	100%	1.143	0.218
Sexual abuse (yes)	Feeling forced	4.4%	5.6%	3.1%	0.237	0.059
	Forcing someone else	20.6%	16.7%	25%	0.720	0.103

^*^p < .05

### Sexuality and Sexual Behavior According to the Availability of Electronic Devices

The intensity of their sexual appetite was slightly lower in those who had electronic devices (42.1%) compared to those who did not have this technology (33%), although these differences did not reach statistical significance (*p* = 0.673; *V* = 0.097) (see Table 6). In general, people who had electronic devices reported higher percentages of masturbation with sex toys (17.5%) and masturbation while viewing pornographic content (25%) than those who did not have these devices (9.1% and 18.2%, respectively). Sexual relations were strongly influenced by the availability of electronic devices. Thus, people without devices reported higher percentages of physical sex (*p* = 0.038; *V* = 0.265), and people with devices reported higher percentages of online sex (*p* = 0.247; *V* = 0.188). In addition, as expected, people with electronic devices engaged in a larger number of online sexual behaviors: sending nude images of oneself (100% vs. 81.3%; *p* = 0.280), viewing pornographic images or movies (87.9% vs. 80%; *p* = 0.366), searching for sexual information (30% vs. 12.1%; *p* = 0.066), and searching for a partner on dating websites (15.3% vs. 9.1%; *p* = 0.445). Finally, the percentage of people who acknowledged having forced themselves on another person was higher in those who had electronic devices (25%), compared to those who did not have this technology (12.1%), although these differences were not statistically significant (*p* = 0.235, *V* = 0.163).

**Table 6:** Sexuality and sexual behavior according to the availability of electronic devices

		Total (*n* = 73)	Yes (*n* = 40)	No (*n* = 33)	*X*^2^	*V*
Intensity of sexual appetite	Less	38%	42.1%	33.3%	0.673	0.097
	The same	23.9%	21.1%	27.3%		
	Greater	38%	36.8%	39.4%		
Masturbation (yes)	Traditional	100%	100%	100%	–	–
	Sex toys	13.7%	17.5%	9.1%	1.082	0.122
	Pornography	21.9%	25%	18.2%	0.791	0.082
Sexual relations with other people (yes)	Physical	5.5%	0%	12.1%	5.130^*^	0.265
	Online	4.1%	7.5%	0%	2.581	0.188
Online sexual behavior (yes)	Search for sexual information	21.9%	30%	12.1%	3.377	0.215
	Watch pornographic films	83.6%	87.9%	80%	0.817	0.106
	Enter dating websites	12.3%	15.3%	9.1%	0.584	0.089
	Send nude images of oneself	88%	100%	81.3%	1.918	0.277
Sexual abuse (yes)	Feeling forced	6.8%	5%	9.1%	0.474	0.081
	Forcing someone else	19.2%	25%	12.1%	1.935	0.163

^*^p < .05

## Discussion

The aim of this study was to analyze the differential impact of the COVID-19 lockdown on the sexual behavior of a group of people with ID, based on their age, gender, marital status, and access to electronic devices. This research is especially relevant given the scarcity of studies designed to analyze the impact of the pandemic on the sexual health of this group (Hough, 2020; Lokot & Avakyan, 2020). This aspect may have been affected by stereotypes that persist and can limit the healthy sexual experiences of people with ID more than in the general population (Gil-Llario et al., 2021; Lam et al., 2021).

Regarding the physical, social, and technological environment where the confinement took place, the results showed that most of the participants were with their families, mainly with parents and siblings. In any case, almost all the people they lived with were people they were related to or lived with regularly. More than half had their own room and access to electronic devices. These data are quite relevant because it is highly likely that the impact of confinement on the experience of sexuality could be influenced by these variables. However, having one’s own room does not necessarily mean having a high level of privacy. In fact, some studies conducted with this population show high levels of supervision by family members and caregivers that limit their independence (Brown & McCann, 2018; Retznik et al., 2021).

The results obtained show that the lockdown influenced sexual desire. The differences were more pronounced depending on the age range, increasing significantly in younger people and decreasing in older people. This finding may seem contradictory, but it is not. In general, young people interact with a larger number of people on a day-to-day basis (Merrells et al., 2019; Wilson et al., 2017), and they have younger parents who, in turn, have more normalizing attitudes about sexuality (Manor-Binyamini & Schreiber-Divon, 2019). Thus, the probability of having a freer sexual experience is significantly higher than in older people. Therefore, it is logical that their sexual appetite increased during the confinement period due to the impossibility of satisfying needs that were probably fulfilled more before confinement. Sexual appetite was also greater in those with a stable partner (with whom they did not live) because the lockdown interfered with the possibility of sharing affection and intimacy like before (Baines et al., 2018; Gil-Llario et al., 2018).

Regarding autoeroticism, our results indicate that masturbation was important for all the individuals evaluated, regardless of gender, age, or other variables. This finding coincides with other studies that conclude that autoeroticism is a common sexual practice of people with ID (Díaz-Rodríguez et al., 2014; Kijak, 2013). In general, women reported a greater use of sex toys for this practice, which was not surprising because most of the sexual accessories available on the market are designed and intended for the female anatomy. Hence, some men only use them with a partner, but not individually (Herbenick et al., 2017). Moreover, our results show higher rates of masturbation while viewing pornography in the youngest participants. Intergenerational changes, along with the greater climate of tolerance towards sexuality in this group, make it easier for young people to have access to and use electronic devices (Darragh et al., 2017). As expected, and as occurred in the general population (Ballester-Arnal et al., 2020), the lockdown could have reduced the possibility of having sexual relations with other people. Only individuals who had a stable partner were able to maintain physical relationships, when the confinement took place in the same physical space, or online relationships through electronic devices when they were confined in different locations.

In addition to autoeroticism, the most frequent sexual behavior, our study also revealed a high percentage of other online sexual behaviors, especially among those who had electronic devices. A large number of studies have found these online behaviors to be a normative element in people with ID (Darragh et al., 2017; Ramsten et al., 2020). In general, online sexual activity can have great benefits when it is carried out to connect with friends or seek information about sexuality. (Chadwick et al., 2017), but it can also have negative consequences in the case of sending nude images, due to the sender’s vulnerability, or viewing pornographic films, due to the risk associated with the poor quality of the models offered in many of them (Chiner et al., 2017). Due to its novelty, the phenomenon of online risks for people with ID is still in an exploratory phase, and the knowledge is still limited (Normand & Sallafranque-St-Louis, 2016). The practice of sexting was one of the most frequent online activities performed during confinement, especially among men. Gómez-Puerta and Chiner (2021) indicate that people with ID perform this practice, even though they are not sufficiently prepared and have not received the necessary training to manage the risks arising from this behavior. In addition to the lack of training and information, there is a lack of supervision by parents or guardians, often due to their low level of digital literacy (Goli et al., 2020).

Finally, the data obtained show that sexual victimization is a phenomenon that has continued to occur during the confinement period. Thus, 6.7% of the people studied felt sexually forced, and 19.2% acknowledged having forced themselves on another person during this period. Undoubtedly, confinement may have favored the presence of this type of violence because, in most cases, the abuse is perpetrated by a member of the immediate family or social environment (Mitra et al., 2016; Tomsa et al., 2021). The abuse rates obtained during confinement are very similar to those reported in other national and international studies that analyze abuse throughout the life cycle, where prevalence ranges from 6 to 9% (Gil-Llario et al., 2018; Mitra et al., 2016). Unfortunately, our data do not allow us to ascertain the degree of severity or whether it involved insistence or consummated abuse. In any case, these data are alarming. Moreover, the number of abuses found could be much higher, given that only a small percentage of all sexual abuses are reported (Willott et al., 2020), due to either shame or lack of awareness and sexual information (Borawska-Charko et al., 2017; Byrne, 2018). In fact, when abuse is reported by a professional, the percentages are four to five times higher than when it is self-reported by the subject (Gil-Llario et al., 2019). The results obtained show that this type of victimization is not mediated by age or the availability of electronic devices, as indicated in other similar studies (Gil-Llario et al., 2018). Furthermore, no gender differences were found in the percentage of people who said they felt forced, but there were differences in the percentage who acknowledged having forced another person, with this figure being much higher in men. In this regard, there is a strong consensus in the scientific literature that, when a person with ID perpetrates sexual abuse, the person responsible is almost always male (Koçtürk & Yüksel, 2021; Wissink et al., 2018).

This study also has some limitations that should be taken into consideration. First, it is a self-reported and retrospective study, which may result in recall bias or social desirability bias when talking publicly about an aspect as intimate as sexuality. In addition, the sample was obtained through convenience sampling. Caution should be used when generalizing some results because this type of sampling does not guarantee representativeness.

## Conclusions

This is one of the first studies carried out with a sample of people with ID, and it has important practical implications. First, it provides information about the impact of COVID-19 confinement on sexual behavior, an aspect that has been studied very little to date, especially in a group whose sexuality is surrounded by numerous myths and false beliefs (Lam et al., 2021). Second, it is evident that sexuality is an intrinsic aspect of people with ID that has continued to be expressed (Borawska-Charko et al., 2017; Turner & Crane, 2016), even though it was modulated by some factors related to the physical, social, and technological environment where the confinement took place. Third, the results reveal that their sexual activity focused on autoeroticism and online behavior, especially viewing pornography and practicing sexting, showing the risks involved in these types of materials and practices without proper training. Finally, this study highlights, once again, that sexual abuse is associated with the family context (Byrne, 2018; Tomsa et al., 2021), given that this lockdown produced episodes of abuse. One of the main practical applications of the results of this study is to identify the aspects that should be addressed as a priority in the prevention of sexual abuse. Affective-sexual education aimed at this group should focus on knowledge of bodily limits and privacy norms. This information could improve the ability to identify abusive behavior perpetrated by people in their immediate environment for whom they feel affection, such as family members or caregivers.

## References

[CR1] American Psychiatric Association (2013). Diagnostic and statistical manual of mental disorders, Fifth Edition (DSM-5).

[CR2] Arnau S (2019). Estudios críticos de y desde la diversidad functional [Critical studies of and from functional diversity].

[CR3] Baines S, Emerson E, Robertson J, Hatton C (2018). Sexual activity and sexual health among young adults with and without mild/moderate intellectual disability. BMC Public Health.

[CR4] Ballester-Arnal R, Nebot-Garcia JE, Ruiz-Palomino E, Giménez-García C, Gil-Llario M (2020). “INSIDE” project on sexual health in Spain: Sexual life during the lockdown caused by COVID-19. Sexuality Research and Social Policy.

[CR5] Bazzo G, Nota L, Soresi S, Ferrari L, Minnes P (2007). Attitudes of social service providers towards the sexuality of individuals with intellectual disability. Journal of Applied Research in Intellectual Disabilities.

[CR6] Borawska-Charko M, Rohleder P, Finlay WML (2017). The sexual health knowledge of people with intellectual disabilities: A review. Sexuality Research and Social Policy.

[CR7] Brown M, McCann E (2018). Sexuality issues and the voices of adults with intellectual disabilities: A systematic review of the literature. Research in Developmental Disabilities.

[CR8] Byrne G (2018). Prevalence and psychological sequelae of sexual abuse among individuals with an intellectual disability: A review of the recent literature. Journal of Intellectual Disabilities.

[CR9] Chadwick DD, Quinn S, Fullwood C (2017). Perceptions of the risks and benefits of internet access and use by people with intellectual disabilities. British Journal of Learning Disabilities.

[CR10] Chiner E, Gómez-Puerta M, Cardona-Moltó MC (2017). Internet use, risks and online behaviour: The view of internet users with intellectual disabilities and their caregivers. British Journal of Learning Disabilities.

[CR11] Darragh, J., Reynolds, L. C., Ellison, C., & Bellon, M. L. (2017). Let’s talk about sex: How people with intellectual disability in Australia engage with online social media and intimate relationships. *Cyberpsychology: Journal of Psychosocial Research on Cyberspace, 11*(1), Article 9. 10.5817/CP2017-1-9

[CR12] Díaz-Rodríguez, I., Gil-Llario, M. D., Ballester-Arnal, R., Morell-Mengual, V., & Molero-Mañes, R. J. (2014). Conocimientos, comportamiento y actitudes sexuales en adultos con discapacidad intellectual [knowledge, attitudes and sexual behavior in adults with intellectual disabilities]. *International Journal of Developmental and Educational Psychology, 3*(1), 415–422. 10.17060/ijodaep.2014.n1.v3.519

[CR13] Franco DG, Cardoso J, Neto I (2012). Attitudes towards affectivity and sexuality of people with intellectual disability. Sexuality & Disability.

[CR14] Frawley P, Wilson NJ (2016). Young people with intellectual disability talking about sexuality education and information. Sexuality & Disability.

[CR15] Gil-Llario MD (2019). La sexualidad de los jóvenes con diversidad funcional intelectual: Derechos y realidades [The sexuality of young people with intellectual functional diversity: Rights and realities]. Revista De Estudios De Juventud.

[CR16] Gil-Llario MD, Morell-Mengual V, Ballester-Arnal R, Díaz-Rodríguez I (2018). The experience of sexuality in adults with intellectual disability. Journal of Intellectual Disability Research.

[CR17] Gil-Llario MD, Morell-Mengual V, Díaz-Rodríguez I, Ballester-Arnal R (2019). Prevalence and sequelae of self-reported and other-reported sexual abuse in adults with intellectual disability. Journal of Intellectual Disability Research.

[CR18] Gil-Llario M, Fernández-García O, Castro-Calvo J, Caballero-Gascón L, Ballester-Arnal R (2021). Validation of a tool to assess attitudes towards sexuality of individuals with intellectual disability (ASEXID): A preliminary study. Sexuality and Disability.

[CR19] Giménez-García, C., Gil-Llario, M. D., Ruiz-Palomino, E., & Díaz-Rodríguez, I. (2017). Abuso sexual y discapacidad intelectual: cómo identifican y valoran la experiencia las personas con discapacidad intelectual y los profesionales que les atienden [Sexual abuse and intellectual disability: How people with intellectual disabilities and their professionals identify and value the experience]. *International Journal of Developmental and Educational Psychology, 4*(1), 129–136. 10.17060/ijodaep.2017.n1.v4.1035

[CR20] Goli, S., Noroozi, M., & Salehi, M. (2020). Parental experiences about the sexual and reproductive health of adolescent girls with intellectual disability: A qualitative study. *Iranian Journal of Nursing and Midwifery Research, 25*(3), 254. 10.4103/ijnmr.IJNMR_258_19 10.4103/ijnmr.IJNMR_258_19 PMC7299411 32724773

[CR21] Gómez-Puerta M, Chiner E (2021). Internet use and online behaviour of adults with intellectual disability: Support workers’ perceptions, training and online risk mediation. Disability & Society.

[CR22] Haydon AA, McRee A, Halpern CT (2011). Unwanted sex among young adults in the United States: The role of physical disability and cognitive performance. Journal of Interpersonal Violence.

[CR23] Herbenick D, Bowling J, Fu T, Dodge B, Guerra-Reyes L, Sanders S (2017). Sexual diversity in the United States: Results from a nationally representative probability sample of adult women and men. PLoS ONE.

[CR24] Hough S (2020). From the editor of sexuality and disability: The impact of COVID-19 on sexuality and Disability—Are we closer or more isolated?. Sexuality and Disability.

[CR25] Hoyos, S., & García, J. M. (2017). La esterilización en las personas con discapacidad cognitiva y psicosocial: una perspectiva crítica a la jurisprudencia constitucional [The sterilization of people with cognitive and psychosocial disabilities: A critical perspective of the constitutional jurisprudence]. *Revista de Derecho Público,* (38). 10.15425/redepub.38.2017.04

[CR26] Hughes K, Bellis MA, Jones L, Wood S, Bates G, Eckley L, McCoy E, Mikton C, Shakespeare T, Officer A (2012). Prevalence and risk of violence against adults with disabilities: A systematic review and meta-analysis of observational studies. Lancet.

[CR27] Jiménez A, Huete A (2013). La discriminación por motivos de discapacidad. Análisis de las respuestas recibidas al cuestionario sobre discriminación por motivos de discapacidad promovido por el CERMI Estatal.

[CR28] Kijak R (2013). The sexuality of adults with intellectual disability in Poland. Sexuality and Disability.

[CR29] Koçtürk N, Yüksel F (2021). Individual and familial characteristics of sexual abuse victims with intellectual disability. Current Psychology.

[CR30] Lafferty A, McConkey R, Taggart L (2013). Beyond friendship: The nature and meaning of close personal relationships as perceived by people with learning disabilities. Disability & Society.

[CR31] Lam A, Yau M, Franklin RC, Leggat PA (2021). Public opinion on the sexuality of people with intellectual disabilities: A review of the literature. Sexuality and Disability.

[CR32] Lokot, M., & Avakyan, Y. (2020). Intersectionality as a lens to the COVID-19 pandemic: Implications for sexual and reproductive health in development and humanitarian contexts. *Sexual and Reproductive Health Matters, 28*(1), article: 1764748. 10.1080/26410397.2020.1764748 10.1080/26410397.2020.1764748 PMC7887979 32366190

[CR33] Manor-Binyamini I, Schreiber-Divon M (2019). Parental perceptions of the sexuality of adolescents with intellectual disabilities. Sexuality and Disability.

[CR34] Martinello E (2014). Reviewing strategies for risk reduction of sexual abuse of children with intellectual disabilities: A focus on early intervention. Sexuality & Disability.

[CR35] Medina-Rico M, López-Ramos H, Quiñónez A (2018). Sexuality in people with intellectual disability: Review of literature. Sexuality & Disability.

[CR36] Merrells J, Buchanan A, Waters R (2019). “We feel left out”: Experiences of social inclusion from the perspective of young adults with intellectual disability. Journal of Intellectual & Developmental Disability.

[CR37] Mitra M, Mouradian VE, Fox MH, Pratt C (2016). Prevalence and characteristics of sexual violence against men with disabilities. American Journal of Preventive Medicine.

[CR38] Morell-Mengual, V., Gil-Llario, M. D., Díaz-Rodríguez, I., & Caballero-Gascón, L. (2017). Actitudes de padres, profesionales y población general hacia la sexualidad de las personas con discapacidad física e intellectual [Attitudes of parents, professionals and general population towards sexuality of people with physical and intellectual disabilities]. *International Journal of Developmental and Educational Psychology, 4*(1), 173–184. 10.17060/ijodaep.2017.n1.v4.1040

[CR39] Morell-Mengual, V., Gil-Llario, M. D., Díaz-Rodríguez, I., Castro-Calvo, J., & Ceccato, R. (2016). *Sexualidad y abuso sexual en hombres y mujeres con discapacidad intelectual* [Sexuality and sexual abuse in men and women with intellectual disabilities]. En J. L. Castejón (Coord.), Psicología y Educación: Presente y Futuro (pp.2873–2879). Asociación Científica de Psicología y Educación.

[CR40] Murphy K, Bantry-White E (2020). Behind closed doors: Human rights in residential care for people with an intellectual disability in Ireland. Disability & Society.

[CR41] Normand CL, Sallafranque-St-Louis F (2016). Cybervictimization of young people with an intellectual or developmental disability: Risks specific to sexual solicitation. Journal of Applied Research in Intellectual Disabilities.

[CR42] Ramsten C, Martin L, Dag M, Hammar LM (2020). Information and communication technology use in daily life among young adults with mild-to-moderate intellectual disability. Journal of Intellectual Disabilities.

[CR43] Retznik, L., Wienholz, S., Höltermann, A., Conrad, I., & Riedel-Heller, S. (2021). “It tingled as if we had gone through an anthill.” Young people with intellectual disability and their experiences with relationship, sexuality and contraception. *Sexuality and Disability*. 10.1007/s11195-020-09670-z

[CR44] Rodríguez, J. M., López, F., Morentin, R., & Arias, B. (2006). Afectividad y sexualidad en personas con discapacidad intelectual. Una propuesta de trabajo [Affectivity and sexuality in people with intellectual disabilities. A work proposal]. Si*glo Cero: Revista Española sobre Discapacidad Intelectual, 37*(217), 23–40.

[CR45] Rogers C (2009). (S)excerpts from a life told: Sex, gender and learning disability. Sexualities.

[CR46] Sinclair J, Unruh D, Lindstrom L, Scanlon D (2015). Barriers to sexuality for individuals with intellectual and developmental disabilities: A literature review. Education and Training in Autism and Developmental Disabilities.

[CR47] Swango-Wilson A (2009). Perception of sex education for individuals with developmental and cognitive disability: A four cohort study. Sexuality & Disability.

[CR48] Tamas D, Jovanovic NB, Rajic M, Ignjatovic VB, Prkosovacki BP (2019). Professionals, parents and the general public: Attitudes towards the sexuality of persons with intellectual disability. Sexuality & Disability.

[CR49] Tomsa R, Gutu S, Cojocaru D, Gutiérrez-Bermejo B, Flores N, Jenaro C (2021). Prevalence of sexual abuse in adults with intellectual disability: Systematic review and meta-analysis. International Journal of Environmental Research and Public Health.

[CR50] Turner GW, Crane B (2016). Pleasure is paramount: Adults with intellectual disabilities discuss sensuality and intimacy. Sexualities.

[CR51] Willott S, Badger W, Evans V (2020). People with an intellectual disability: Under-reporting sexual violence. The Journal of Adult Protection.

[CR52] Wilson NJ, Jaques H, Johnson A, Brotherton ML (2017). From social exclusion to supported inclusion: Adults with intellectual disability discuss their lived experiences of a structured social group. Journal of Applied Research in Intellectual Disabilities.

[CR53] Wissink IB, Vugt ESV, Smits IAM, Moonen XMH, Stams GJM (2018). Reports of sexual abuse of children in state care: A comparison between children with and without intellectual disability. Journal of Intellectual & Developmental Disability.

[CR54] World Health Organization [WHO]. (2021). Violence against adults and children with disabilities. https://www.who.int/disabilities/violence/en/

